# Bringing precision medicine to the management of pregnancy in women with glucokinase-MODY: a study of diagnostic accuracy and feasibility of non-invasive prenatal testing

**DOI:** 10.1007/s00125-023-05982-9

**Published:** 2023-08-31

**Authors:** Alice E. Hughes, Jayne A. L. Houghton, Benjamin Bunce, Ali J. Chakera, Gill Spyer, Maggie H. Shepherd, Sarah E. Flanagan, Andrew T. Hattersley

**Affiliations:** 1 Faculty of Health and Life Sciences, University of Exeter Medical School, Royal Devon and Exeter NHS Foundation Trust, Exeter, UK; 2 Exeter Genomics Laboratory, Royal Devon University Healthcare NHS Foundation Trust, Exeter, UK; 3 Department of Diabetes and Endocrinology, Royal Sussex County Hospital, University Hospitals Sussex NHS Foundation Trust, Brighton, UK; 4 Department of Diabetes and Endocrinology, Torbay Hospital, Torbay and South Devon NHS Foundation Trust, Torquay, UK; 5 National Institute for Health and Care Research, Exeter Clinical Research Facility, Royal Devon University Healthcare NHS Foundation Trust, Exeter, UK

**Keywords:** Diabetes pregnancy, Glucokinase, Maturity-onset diabetes of the young, MODY, Non-invasive prenatal testing, Precision medicine, Ultrasound

## Abstract

**Aims/hypothesis:**

In pregnancies where the mother has glucokinase-MODY (GCK-MODY), fetal growth is determined by fetal genotype. When the fetus inherits a maternal pathogenic *GCK* variant, normal fetal growth is anticipated, and insulin treatment of maternal hyperglycaemia is not recommended. At present, fetal genotype is estimated from measurement of fetal abdominal circumference on ultrasound. Non-invasive prenatal testing of fetal *GCK* genotype (NIPT-GCK) using cell-free DNA in maternal blood has recently been developed. We aimed to compare the diagnostic accuracy of NIPT-GCK with that of ultrasound, and determine the feasibility of using NIPT-GCK to guide pregnancy management.

**Methods:**

We studied an international cohort of pregnant women with hyperglycaemia due to GCK-MODY. We compared the diagnostic accuracy of NIPT-GCK with that of measurement of fetal abdominal circumference at 28 weeks’ gestation (*n*=38) using a directly genotyped offspring sample as the reference standard. In a feasibility study, we assessed the time to result given to clinicians in 43 consecutive pregnancies affected by GCK-MODY between July 2019 and September 2021.

**Results:**

In terms of diagnostic accuracy, NIPT-GCK was more sensitive and specific than ultrasound in predicting fetal genotype (sensitivity 100% and specificity 96% for NIPT-GCK vs sensitivity 53% and specificity 61% for fetal abdominal circumference 75th percentile). In terms of feasibility, a valid NIPT-GCK fetal genotype (≥95% probability) was reported in all 38 pregnancies with an amenable variant and repeated samples when needed. The median time to report was 5 weeks (IQR 3–8 weeks). For the 25 samples received before 20 weeks’ gestation, results were reported at a median gestational age of 20 weeks (IQR 18–24), with 23/25 (92%) reported before 28 weeks.

**Conclusions/interpretation:**

Non-invasive prenatal testing of fetal genotype in GCK-MODY pregnancies is highly accurate and is capable of providing a result before the last trimester for most patients. This means that non-invasive prenatal testing of fetal genotype is the optimal approach to management of GCK-MODY pregnancies.

**Graphical Abstract:**

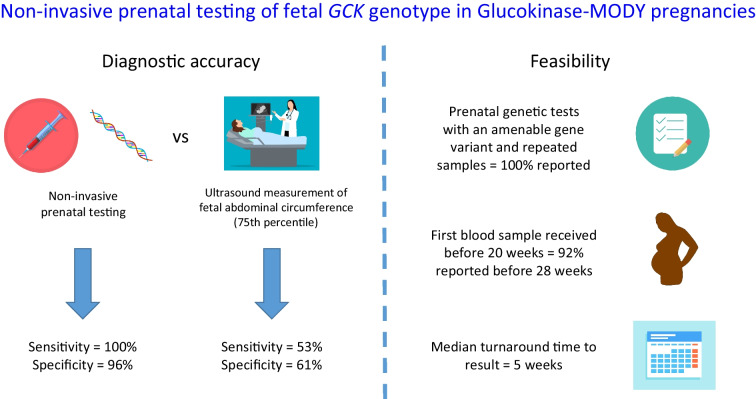

**Supplementary Information:**

The online version of this article (10.1007/s00125-023-05982-9) contains peer-reviewed but unedited supplementary material.


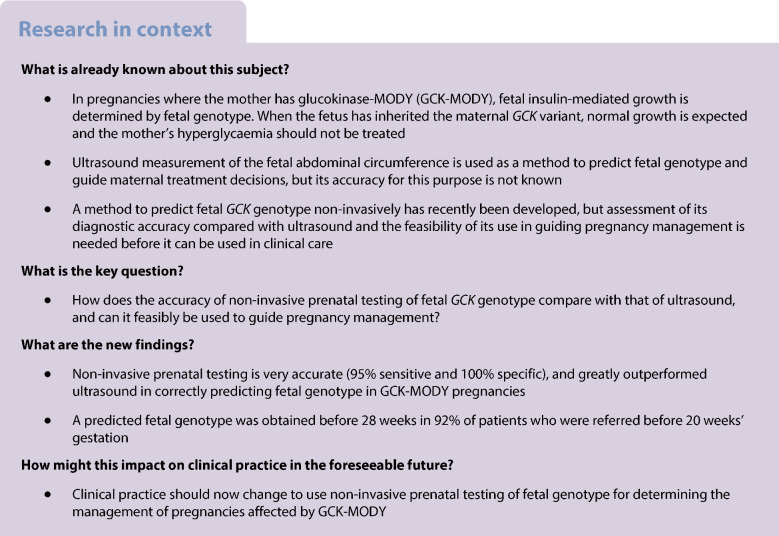


## Introduction

Heterozygous inactivating variants in the glucokinase gene (*GCK*) result in MODY (GCK-MODY) [1]. GCK-MODY is the commonest form of MODY, affecting approximately 1 in 1000 people [2]. It is characterised by a mild fasting hyperglycaemia (5.5–8 mmol/l) from birth, which is not associated with long-term complications and does not require treatment outside of pregnancy [3].

In the third trimester, insulin is a key intrauterine growth factor [4], and is secreted by the fetus in response to maternal blood glucose that crosses the placenta into the fetal circulation [5]. Offspring of mothers with high blood glucose during pregnancy are at risk of being large for gestational age (LGA; birthweight >90th percentile for sex and gestational age) as they secrete more insulin in response to the prevailing high blood glucose [6].

In GCK-MODY pregnancies, the insulin secretory response to maternal hyperglycaemia is dependent on the fetal genotype [7]. When the *GCK* variant has not been inherited, the fetus senses the maternal glucose as being high and secretes insulin. These fetuses are consequently born 500–600 g heavier than mean birthweight, with a high risk of being LGA and macrosomic [8–12]. Conversely, fetuses who inherit a maternal *GCK* variant do not sense the maternal glucose levels as being high, and secrete insulin at a similar threshold to their mother. This results in normal birthweight, with LGA rates that are comparable with those of the general population [8–11].

Treatment of maternal hyperglycaemia is also dependent on the fetal genotype, with insulin treatment not being necessary or desirable when the fetus has inherited the *GCK* variant and is predicted to be of normal birthweight. There is also evidence that insulin treatment restricts fetal growth in this scenario, increasing the risk of the baby being small for gestational age (birthweight <10th percentile) [8, 9, 12, 13]. When the fetus has not inherited the maternal *GCK* variant, treatment of maternal hyperglycaemia with insulin may reduce the risk of the baby being LGA [8, 11, 12]. Therefore, prenatal identification of fetal genotype is critical to help guide management of GCK-MODY pregnancies.

A widely used approach to predict fetal genotype takes advantage of the differences in fetal growth observed between fetuses who do and do not inherit a maternal *GCK* variant [1, 8, 14, 15]. Fetuses at higher risk of LGA, which is the case where a fetus has not inherited a maternal *GCK* variant, have a larger abdominal circumference [16]. Use of a threshold of >75th percentile to detect a fetus who has not inherited a maternal *GCK* variant was established from trials of ultrasound-guided management of mild gestational diabetes [17, 18]. A threshold >90th percentile has also been proposed to indicate the presence of a fetus who has not inherited the maternal *GCK* variant [14, 19, 20]. However, the precision of ultrasound and these thresholds for classifying fetal genotype has not been studied in GCK-MODY pregnancies. Furthermore, insulin-mediated fetal growth does not become apparent until the third trimester [4], limiting its use in guiding management until later in pregnancy [21].

Direct fetal genotyping by invasive testing using chorionic villous sampling or amniocentesis is possible when carried out for another diagnostic indication [14]. This is clearly an accurate guide of fetal genotype. However, it cannot be used as routine practice due to the slight risk of fetal loss [22]. This has led to the need to develop a non-invasive methodology using cell-free DNA.

Analysis of cfDNA in maternal blood has revolutionised some areas of prenatal genetic testing, notably for aneuploidy [23], by offering a non-invasive and therefore safer approach to screening of fetal genetic disease. Detecting a heterozygous fetal genotype in the setting of a heterozygous mother (as is the case with GCK-MODY pregnancy) is challenging, and although some methods are now available, they are still not widely implemented.

Methods to predict fetal *GCK* genotype non-invasively have recently been developed based on relative mutation dosage and relative haplotype dosage [24–26]. These methods have provided accurate results in wholly retrospective analyses on limited numbers of samples. They have not been introduced clinically or assessed in a prospective real-time clinical study in comparison with the current practice of ultrasound. A prospective study is needed before clinical implementation.

In this study, we aimed to assess the accuracy of non-invasive prenatal testing in predicting fetal genotype compared with that of an ultrasound scan, and to determine the feasibility of this approach in management of pregnancies where the mother has GCK-MODY.

## Methods

### Study design and participants

**Fig. 1:**
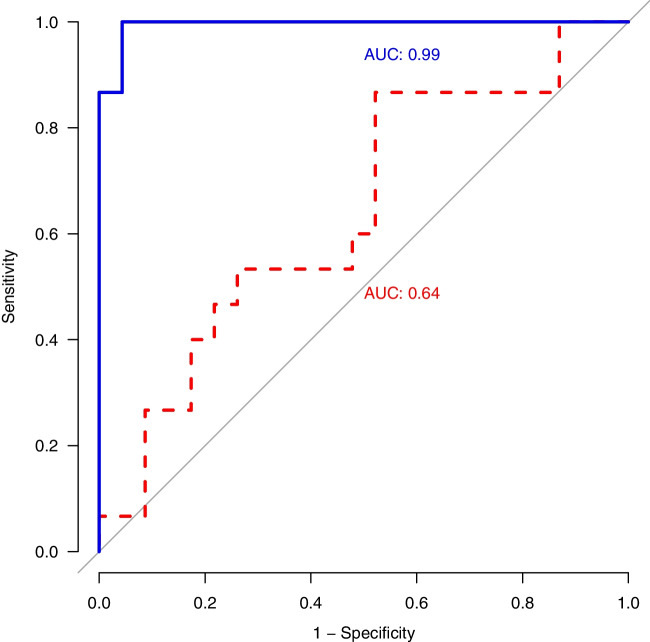
ROC curves showing the AUC for the probability of N/M fetal genotype based on NIPT compared with an abdominal circumference percentile measurement on ultrasound at 28 weeks’ gestation (*p*<0.0001). The results for NIPT are indicated by the blue solid curve; those for ultrasound are indicated by the red dashed curve. The ROC curves are paired (contain 38 test results each)

Two aligned studies were performed with overlapping participants (summarised in electronic supplementary material (ESM) Fig. 1). The first study assessed the diagnostic accuracy of non-invasive prenatal testing (NIPT) compared with that of a 28-week ultrasound scan in predicting fetal *GCK* genotype using a directly genotyped offspring sample as the reference standard. The second study prospectively determined the feasibility of non-invasive testing in guiding the management of GCK-MODY pregnancies in 43 cases. The full study protocol can be found at https://www.diabetesgenes.org/current-research/gck-mody-nipt/. All participants gave informed written consent for testing and collection of clinical data as part of entry to the Genetic Beta Cell Research Bank (https://www.diabetesgenes.org/current-research/genetic-beta-cell-research-bank/). Ethics approval was given by the North Wales Research Ethics Committee (Multi-centre Research Ethics Committee number 17/WA/0327). Participants were referred from across the world to the Exeter Genomics Laboratory for genetic testing. Participants were of reproductive age and the majority originated from the UK and were of white self-reported ethnicity. Information on gender and regional and socioeconomic factors was not available.

#### Diagnostic accuracy study

We obtained data from 38 pregnancies of 38 women with GCK-MODY diagnosed before or during pregnancy for which both NIPT samples and an ultrasound at 26–30 weeks’ gestational age were available as well as a reference standard direct genetic test of fetal genotype (ESM Table 1).

#### Feasibility study

We prospectively studied 43 pregnancies referred for non-invasive testing of fetal *GCK* genotype between July 2019 and October 2021 (ESM Table 2). There was an overlap of 23 pregnancies between the feasibility and diagnostic accuracy studies (ESM Fig. 1).

### Non-invasive prenatal testing methodology

We performed NIPT as previously described [24]. Briefly, cfDNA was extracted from maternal plasma of venous blood samples and analysed using digital-droplet PCR. Specificity of the assay used for the relevant *GCK* variant with 50:50 allelic balance was verified using maternal genomic DNA. Where the sex of the offspring was known to be male, fetal fraction was determined using X-linked or Y-linked genes (*ZFX* and *ZFY*, respectively). Where sex was unknown, informative SNPs were identified from parental genomic DNA or massively parallel sequencing of cfDNA. A Bayesian Markov chain Monte Carlo analysis using raw data from the digital-droplet PCR of cfDNA was used to predict the probability of fetal genotype. Specificity of the assay for the specified *GCK* variant, fetal fraction >2% and paternal allele droplet count >10 were required for analysis of the digital-droplet PCR data and valid probability (≥95%) of fetal genotype. When an initial sample failed quality control, a further maternal cell-free sample was requested and analysed.

Where the probability of fetal *GCK* heterozygosity was ≥95%, the fetus was predicted to have inherited the maternal *GCK* variant (N/M), and where the probability of fetal *GCK* heterozygosity was ≤5%, the fetus was predicted to have not inherited the maternal *GCK* variant (N/N). These cut-offs agree with the recommended cut-off in the validation phase of the testing method [24]. We included only one sample per pregnancy if more than one was tested, using the earliest available sample with a reportable result (*n*=11).

We performed Sanger sequencing to confirm offspring genotype using umbilical cord or venous blood, buccal swab, chorionic villous or amniotic fluid samples. Primer and probe sequences are available on request.

### Ultrasound measurements of the fetal abdominal circumference

Ultrasounds were performed by ultrasonographers as part of routine pregnancy care. A gestational age window of 26 to 30 weeks was chosen as scans used to monitor fetal growth in diabetes pregnancies typically start at approximately 28 weeks’ gestation [27]. When more than one ultrasound was performed between 26 and 30 weeks’ gestation (*n*=5), the one performed closest to 28 weeks was used. If there were two scans that were equidistant, the earliest was used. The INTERGROWTH-21st fetal growth standards were used to calculate abdominal circumference percentile for exact gestational age of measurement [28].

Thresholds of <75th and <90th percentile for gestational age were used to determine ultrasound test positivity (i.e. a fetus predicted to have inherited the maternal *GCK* variant), in line with existing recommendations [14, 19, 20].

### Statistical analyses

All statistical analyses were performed using StataSE 17 software (StataCorp, TX, USA) or R version 4.0.3 software [29]. Data plots were generated using the ggplot2 package [30]. An *α* of 0.05 was considered statistically significant, and all *p* values were obtained using two-sided tests. Participant characteristics were summarised and continuous data were compared using the Wilcoxon rank-sum test, and counts were compared using Fisher’s exact test.

#### Diagnostic accuracy study analyses

A minimum sample size of 35 was required to detect a 23% difference in probability of correctly identifying a fetus who had inherited the maternal *GCK* variant between NIPT for fetal *GCK* genotype (NIPT-GCK) and an abdominal circumference <75th percentile with 80% power at *α* = 0.05 (see study protocol [https://www.diabetesgenes.org/current-research/gck-mody-nipt/] for details) [31].

We compared measures of accuracy (sensitivity, specificity, positive predictive value [PPV] and negative predictive value [NPV]) between NIPT-GCK and ultrasound. We calculated binomial 95% CIs and compared sensitivity and specificity using McNemar’s test [32] and PPV and NPV using a generalised score statistic [33], implemented in the R package DTComPair [34]. Receiver operating characteristic (ROC) curves were generated using the fetal abdominal circumference percentiles and non-invasive prenatal test probabilities, and compared using the bootstrap method implemented in the R package pROC [35]. This comparison also showed that the sample size of 38 pregnancies had 83% power to determine a difference in AUC at α = 0.05.

#### Feasibility study analyses

Continuous data were compared using the Wilcoxon rank-sum test. Correlations between gestational age of receipt of the first maternal blood sample and time to result were tested using Spearman’s rho.

## Results

### Diagnostic accuracy

We studied diagnostic accuracy in 38 eligible pregnancies for which a reportable non-invasive prenatal test, a 28-week ultrasound and a confirmed offspring genotype were available. The characteristics of the women included in this study are shown in ESM Table 1.

#### Non-invasive prenatal testing is highly predictive of fetal genotype and more accurate than using ultrasound scans

NIPT produced a result that was concordant with offspring genotype in 37/38 pregnancies (97%) (Table 1), and showed excellent sensitivity (100%; 95% CI 78, 100%), specificity (96%, 95% CI 78, 100%), PPV (94%, 95% CI 70, 100%) and NPV (100%, 95% CI 85, 100%).

**Table 1:** Performance of NIPT (NIPT) for diagnosis of fetal *GCK* genotype

		Fetal genotype		
		Confirmed N/M	Confirmed N/N	
NIPT test result^a^	Predicted N/M	15	1	PPV 94% (70, 100%)
	Predicted N/N	0	22	NPV 100% (85, 100%)
		Sensitivity 100% (78, 100%)	Specificity 96% (78, 100%)	

Measures of accuracy are accompanied by exact binomial 95% CIs in parentheses

^a^A predicted N/M test result refers to a ≥95% probability that a fetus has inherited the maternal GCK-MODY variant; a predicted N/N test result refers to a ≤5% probability that the fetus has inherited the maternal GCK-MODY variant

N/M indicates the presence of a *GCK* variant; N/N indicates that no *GCK* variant is present

In contrast, the 75th percentile abdominal circumference threshold on 28-week ultrasound was only concordant with offspring genotype in 22/38 pregnancies (58%) (Table 2), with lower sensitivity (53%, 95% CI 27, 79%), specificity (61%, 95% CI 39, 80%), PPV (47%, 95% CI 23, 72%) and NPV (67%, 95% CI 43, 85%) compared with non-invasive testing.

**Table 2:** Performance of fetal abdominal circumference (75th percentile threshold) measured by ultrasound at 28 weeks’ gestation (range 26–30) for diagnosis of fetal *GCK* genotype

		Fetal genotype		
		Confirmed N/M	Confirmed N/N	
Ultrasound test result^a^	Predicted N/M	8	9	PPV 47% (23, 72%)
	Predicted N/N	7	14	NPV 67% (43, 85%)
		Sensitivity 53% (27, 79%)	Specificity 61% (39, 80%)	

Measures of accuracy are accompanied by exact binomial 95% CIs in parentheses

^a^A predicted N/M test result refers to an abdominal circumference measurement that is <75th percentile for gestational age, and a predicted N/N test result refers to an abdominal circumference measurement that is >75th percentile for gestational age, both according to the INTERGROWTH-21st standards [27]

N/M indicates the presence of a *GCK* variant; N/N indicates that no *GCK* variant is present

Using the 90th percentile abdominal circumference threshold resulted in 24/38 pregnancies (63%) being concordant with offspring genotype (Table 3). Compared with the 75th centile threshold, this method showed improved sensitivity (87%, 95% CI 60, 98%), but with reduced specificity (48%, 95% CI 27, 69%) and PPV and NPV of 52% (95% CI 31, 72%) and 85% (95% CI 55, 98%), respectively.

**Table 3:** Performance of fetal abdominal circumference (90th percentile threshold) measured by ultrasound at 28 weeks’ gestation (range 26–30) for diagnosis of fetal *GCK* genotype

		Fetal genotype		
		Confirmed N/M	Confirmed N/N	
Ultrasound test result^a^	Predicted N/M	13	12	PPV 52% (31, 72%)
	Predicted N/N	2	11	NPV 85% (55, 98%)
		Sensitivity 87% (60, 98%)	Specificity 48% (27, 69%)	

Measures of accuracy are accompanied by exact binomial 95% CIs in parentheses

^a^A predicted N/M test result refers to an abdominal circumference measurement that is <90th percentile for gestational age, and a predicted N/N test result refers to an abdominal circumference measurement that is >90th percentile for gestational age, both according to the INTERGROWTH-21st standards [27]

N/M indicates the presence of a *GCK* variant; N/N indicates that no *GCK* variant is present

Continuous test measures from these 38 pregnancies showed NIPT-GCK to be more accurate than ultrasound (Fig. 1; ROC AUC 0.99 [95% CI 0.97, 1.00] vs 0.64 [95% CI 0.46, 0.83], *p*<0.0001). Based on this comparison, the fetal abdominal circumference threshold that optimised both sensitivity and specificity for detecting an affected fetal genotype was the 91st percentile (sensitivity 87%, specificity 48%), which is close to the previously suggested 90th percentile.

### Feasibility study

**Fig. 2:**
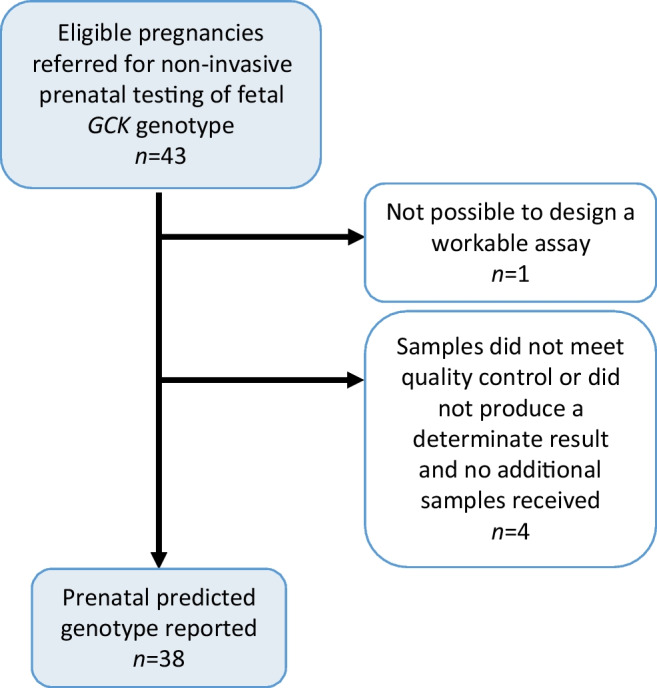
Flow chart of pregnancies included in the feasibility study of use of NIPT for determining fetal *GCK* genotype

We assessed the feasibility of providing physicians with results of NIPT of fetal genotype in 43 consecutive pregnancies (Fig. 2). The characteristics of the women included in this study are shown in ESM Table 2.

#### Receiving a result during the pregnancy

A result from NIPT-GCK was issued to the referring clinician in 38/43 pregnancies (88%). Obtaining a result was not possible in one pregnancy because an assay could not be designed for their variant, and in four pregnancies because the initial samples did not meet quality control standards (*n*=1) or the reporting threshold (*n*=3) and additional samples were not received.

#### Non-invasive prenatal testing can provide a predicted fetal genotype prior to the start of growth scans

 The median gestational age at the time of issuing the genetic report result was 25 weeks (IQR 19–30). In the 25 pregnancies for which the first sample was received prior to 20 weeks, the median gestational age for the result was 20 weeks (IQR 18–24), and 92% of these results (23/25) were reported before 28 weeks’ gestation.

#### Factors influencing turnaround time for getting a non-invasive genotype result

The median turnaround time from receipt of all first samples to a result was 5 weeks (IQR 3–8). There was a shorter turnaround time in the 27/38 women (71%) for whom only one blood sample was required (median 4 weeks [IQR 2–5] vs 12 weeks [IQR 10–14] for those who required more than one sample; *p*<0.0001). The nine women (24%) who had a pre-existing assay for the patient-specific *GCK* variant also had a shorter turnaround time (median 2 weeks [IQR 2–3] vs 7 weeks [IQR 4–11]; *p* = 0.00018).

Information on pregnancy management and outcomes for pregnancies for which a result was received was available for 26 pregnancies and is summarised in ESM Tables 3 and 4.

## Discussion

### Statement of principal findings

**Fig. 3:**
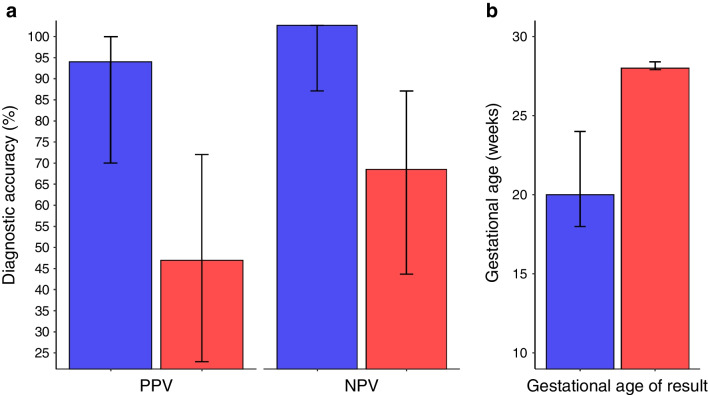
Summary comparison of NIPT vs ultrasound at 28 weeks’ gestation. (**a**) Comparison of diagnostic accuracy (PPV and NPV) between NIPT (blue bars) and an ultrasound scan (red bars) using a fetal abdominal circumference threshold of the 75th percentile (data from Tables 1 and 2, *n*=38). (**b**) Comparison of the median time at which a result was obtained for individuals who had a result reported by NIPT (blue bars) when referred before 20 weeks in the feasibility study and the median gestational age of the scan (red bars) from the diagnostic accuracy study. The error bars show the 95% CIs for the PPV and the NPV, and the IQR for gestational age of diagnosis

This study in GCK-MODY pregnancies has shown that NIPT markedly improves the prediction of fetal genotype compared with the current clinical practice of using fetal abdominal circumference on ultrasound (Fig. 3). Not only are the results more accurate, with the PPV being >40% higher, but also these results can be obtained earlier, usually before the onset of fetal insulin-mediated growth at approximately 24 weeks’ gestation [36]. This means that NIPT-GCK can facilitate a more tailored approach to pregnancy management in mothers with GCK-MODY, ensuring that insulin treatment is not started when the fetus has inherited the mutation.

NIPT-GCK was highly accurate, with 100% of fetuses who had inherited the maternal *GCK* variant and 96% of fetuses who had not inherited the maternal *GCK* variant being correctly classified. We obtained one false-positive result despite meeting the strict quality control and analytical criteria. However, as we specify a reportable result as being a probability of fetal genotype ≥ 95%, the discordant result rate of 3% (1/38) in the pregnancies analysed here falls within the expected margin of error.

A key aspect of our study is that, to our knowledge, it is the first study to compare NIPT with the presently used method of ultrasound assessment of fetal abdominal circumference at 28 weeks. Use of this fetal ultrasound assessment takes advantage of the fact that, when the fetus does not inherit a maternal *GCK* variant, there is increased fetal growth with an increased abdominal circumference due to higher fetal insulin secretion [7, 37]. However, use of the 28-week ultrasound was markedly less accurate than using NIPT: using the 90th percentile of the fetal abdominal circumference, which was slightly more discriminatory than the 75th percentile, resulted in only 87% of affected fetuses and 48% of unaffected fetuses being correctly diagnosed.

We were also able to demonstrate in the prospective feasibility study that the results of NIPT could be obtained sufficiently rapidly during pregnancy to play a key role in management. We showed that, when the first sample was received by the laboratory before 20 weeks’ gestation, a result was obtainable at a median gestational age of 20 weeks, with 92% receiving a result before 28 weeks. This means that early referral for NIPT will usually result in accurate prediction of fetal genotype before the 28-week growth scan. This is useful as it means that a decision on whether to use insulin treatment in the pregnancy can be made earlier.

The question of whether routine monitoring of fetal growth should continue to be performed if the fetus is predicted to have inherited the maternal variant by NIPT has not yet been answered. The evidence of benefit for growth scans to detect macrosomia in women without gestational diabetes is weak [38, 39] and is not routine practice, suggesting it is unlikely to be required when the fetus has inherited the *GCK* variant. However, ultrasound could be beneficial in helping plan the mode and timing of delivery when the fetus has not inherited the maternal *GCK* variant, as it may help to identify fetuses at higher risk for being born LGA [16].

### Strengths and weaknesses of the study

The major strengths of these studies are that they address the two key questions that need to be answered before the adoption of NIPT-GCK into clinical care: is it more accurate than the present ultrasound method and is it possible to get a result back sufficiently rapidly during the pregnancy? One limitation of our accuracy study is that it is of limited size (*n*=38), but studies of larger cohorts will be difficult as GCK pregnancies are rare and often not detected [1]. The method that we describe for NIPT also will not work for all pathogenic variants, as it cannot test large insertions/deletions and copy-number variants, which are responsible for approximately 10% of variants causing GCK-MODY [40]. Finally, although this method can give an early result in women who are known to have GCK-MODY prior to pregnancy, it is unlikely that NIPT may guide pregnancy management in women who are first identified as having GCK-MODY in pregnancy, particularly when it is detected following oral glucose tolerance testing at 26–28 weeks.

### Strengths and weaknesses in relation to other studies

Our previous study of the NIPT method used in this study showed that it was accurate at diagnosing fetal genotype during pregnancy in principle using 42 previously collected samples from 29 pregnancies [24]. No comparison was made with ultrasound and feasibility was not assessed. Two other studies of an alternative method using relative haplotype dosage also showed a high level of concordance with a postnatal confirmed genotype, but these were limited to five cases and were performed on historically collected samples only [25, 26]. The relative haplotype dosage method is likely to be as good as relative mutation dosage (the method studied here) for prospective diagnosis, but similar studies of implementation in clinical practice are needed.

### Implications of the study for clinicians and policymakers

This result is important for clinicians looking after mothers with known GCK-MODY in pregnancy, as NIPT-GCK much more accurately classifies fetal genotype compared with ultrasound, and this will help guide management. A more accurate prediction of fetal genotype with non-invasive testing may prevent iatrogenic harm, as maternal insulin treatment has been associated with a higher risk of being small for gestational age for GCK-MODY babies who inherit their mother’s *GCK* variant [9]. As babies who are small for gestational age are at higher risk of morbidity and mortality [41], the impact of non-invasive testing in this context is important. It will be important to make this testing widely available.

The major limitation to the use of NIPT in this situation is the cost, which was found to be approximately £2000 in a study published in 2016 [42]. However, the UK National Health Service recently formally accredited use of this test in GCK-MODY pregnancies following an appraisal of the evidence comparing its performance with ultrasound.

### Unanswered questions and future research

The key area for future research now an accurate non-invasive method has been developed to determine fetal genotype prenatally is to refine maternal management when fetal genotype is known. It is not clear whether treatment of maternal hyperglycaemia reduces excess fetal growth when the fetus has not inherited the maternal variant [8–11, 13], nor is it clear when glucose monitoring and treatment should begin in pregnancy. Use of NIPT has the potential to answer questions about optimal pregnancy management in a trial setting.

NIPT-GCK may also play a role in predicting babies who are at risk of neonatal hypoglycaemia, as it is unlikely to occur in babies who have inherited their mother’s *GCK* variant. We did observe two cases of neonatal hypoglycaemia requiring intensive or special neonatal care, and these both occurred in pregnancies where the baby did not have GCK-MODY. However, this study was not designed or powered to determine whether use of NIPT affected pregnancy outcomes.

### Concluding remarks

In conclusion, NIPT in pregnancies where the mother has GCK-MODY can provide a more accurate prediction of fetal genotype than use of ultrasound. It is possible to obtain a result prior to the third trimester, which can enable a focused approach to pregnancy management, preventing or stopping unnecessary and potentially harmful insulin treatment where the fetus has inherited the maternal variant.

### Supplementary Information

Below is the link to the electronic supplementary material.

Supplementary file1 (PDF 197 KB)

## Data Availability

Individual-level data used in this study are not freely available to protect the identity of research participants. Information for researchers and protocols for the study and submissions to the Genetic Beta Cell Research Bank are available online (https://www.diabetesgenes.org/current-research/gck-mody-nipt/ and https://www.diabetesgenes.org/current-research/genetic-beta-cell-research-bank/). Requests for additional data specifically related to this work are available to researchers through managed open collaboration. Requests will be considered after liaising with the relevant study and ethics committees, and should be made in writing to the corresponding author, AT Hattersley.

## Abbreviations

AC: Abdominal circumference
GCK: Glucokinase
LGA: Large for gestational age
NIPT: Non-invasive prenatal testing
NIPT-GCK: Non-invasive prenatal testing for fetal *GCK* genotype
NPV: Negative predictive value
PPV: Positive predictive value
ROC: Receiver operating characteristic
