# Supplementary material for: Bringing precision medicine to the management of pregnancy in women with glucokinase-MODY: a study of diagnostic accuracy and feasibility of non-invasive prenatal testing
Source: Diabetologia. 2023 Aug 31;66(11):1997–2006. doi: 10.1007/s00125-023-05982-9 (PMC10542291; doi:10.1007/s00125-023-05982-9)
Supplement: Supplementary file 1 — Supplementary file1 (PDF 197 KB) [file 125_2023_5982_MOESM1_ESM.pdf]

Electronic Supplementary Material

ESM Table 1. Characteristics of the 38 pregnancies and samples included in the diagnostic accuracy study. Continuous data are shown as medians with interquartile range (IQR) and categorical data are shown as counts with percentages.

	**Median (IQR) or count (%)**
**Characteristic**	
Mother's age, years	32 (29–35)
Missing	0 (0%)
Mother's pre or early pregnancy BMI, kg/m^2	23.5 (21.7–26.9)
Missing	0 (0%)
Mother's self-reported ethnicity	
White	35 (92%)
Hispanic	1 (3%)
Not known	2 (5%)
Mother's parity	
0	17 (45%)
1	14 (37%)
>1	7 (18%)
Missing	0 (0%)
GCK-MODY diagnosis known before pregnancy	24 (63%)
Missing	0 (0%)
Fasting plasma glucose at diagnosis of GCK-MODY, mmol/L	6.4 (6.2–7.0)
Missing	11 (29%)
Gestational age of ultrasound scan included, weeks	28 (27.9–28.3)
Missing	0 (0%)
Mother received insulin treatment at the time of ultrasound scan	
Yes	17 (45%)
No	21 (55%)
Number of samples tested	
1	27 (71%)
2	9 (24%)
3	2 (5%)
Number of samples meeting quality control	
1	32 (84%)
2	4 (11%)
3	2 (5%)
Number of samples needed for reportable result[a]	
1	30 (79%)
2	7 (18%)
3	1 (3%)
Gestational age of sample included, weeks[†]	21 (13–28)

[a]For samples tested in the validation phase of the method, samples of a later gestational age were tested in two pregnancies, but a sample of an earlier gestational age produced a reportable result.

ESM Table 2. Characteristics of the 43 pregnancies and samples included in the feasibility study. Continuous data are shown as medians with interquartile ranges (IQR) and categorical data are shown as counts with percentages.

Characteristic	Median (IQR) or count (%)
Mother's age, years	31 (27–36)
Missing	0 (0%)
Mother's pre- or early pregnancy BMI, kg/m^2	24.0 (21.0–27.1)
Missing	1 (2%)
Mother's self-reported ethnicity	
White	35 (81%)
Hispanic	1 (2%)
Asian	4 (9%)
Not known	3 (7%)
Mother's parity	
0	22 (51%)
1	14 (33%)
>1	5 (12%)
Missing	2 (5%)
GCK-MODY diagnosis known before pregnancy	29 (67%)
Missing	0 (0%)
Fasting plasma glucose at diagnosis of GCK-MODY, mmol/L	6.8 (6.3–7.1)
Missing	16 (37%)
Number of samples tested	
1	31 (72%)
2	10 (23%)
3	2 (5%)
Number of samples meeting quality control	
0	1 (2%)
1	36 (84%)
2	4 (9%)
3	2 (5%)
Number of samples needed for a reportable result	
1	27 (63%)
2	9 (21%)
3	2 (5%)
Not applicable[a]	5 (12%)
Predicted fetal genotype	
N/M	20 (47%)
N/N	18 (42%)
Not applicable	5 (12%)
Confirmed offspring genotype	
N/M	16 (37%)
N/N	20 (47%)
Not available	7 (16%)
Eligible for diagnostic accuracy study	23 (53%)

[a]We were not able to design a workable assay in one pregnancy and we did not receive additional samples prior to delivery in four pregnancies.

ESM Table 3. Pregnancy management where a fetal genotype was reported to the clinician.

	**Predicted fetal genotype N/N (*n*=12)**	**Predicted fetal genotype N/M (*n*=14)[a]**
Maternal treatment with insulin at the time of receiving NIPT result (%)	5 (42%)	4 (29%)
Maternal treatment with insulin at delivery (%)	5 (42%)	1 (7%)

NIPT=Non-invasive prenatal testing.
[a]Two fetuses predicted to have inherited the maternal *GCK* mutation did not have their genotype confirmed after birth.

ESM Table 4. Offspring outcomes where a fetal genotype was reported to the clinician.

	**Confirmed offspring genotype N/N (*n*=13)[a]**	**Confirmed offspring genotype N/M (*n*=11)**	**Unconfirmed offspring genotype (*n*=2)[b]**
Median gestational age of delivery (IQR)	38.9 (38.1–39.4)	39.3 (37.7–40.0)	37.6 (37.4–37.9)
Median birth weight Z score[c]	0.88 (-0.25–1.50)	0.09 (-0.71–1.24)	0.39 (0.06–0.73)
Birthweight LGA[d] (%)	5 (38%)	2 (18%)	0 (0%)
NICU/SCBU admission for neonatal hypoglycaemia (%)	2 (15%)	0 (0%)	0 (0%)

LGA=large for gestational age, NICU=Neonatal Intensive Care Unit, SCBU=Special Care Baby Unit.
[a]The baby confirmed not to have inherited the maternal variant (false positive NIPT result) is included within the pregnancy outcomes by their confirmed genotype. They were not exposed to maternal treatment, were born LGA and did not have neonatal hypoglycaemia.
[b]These two babies were predicted to have inherited the maternal *GCK* mutation.
[c]Population-based Z score for sex and gestational age at birth using the 1990 UK-WHO standards [2].
[d]Defined as a birthweight >90th percentile for sex and gestational age according to the 1990 UK-WHO standards [2].

ESM Figure 1. Outline of pregnancies and non-invasive prenatal testing samples that contributed to the diagnostic accuracy and feasibility studies. Additional details relating to the pregnancies and samples are in ESM Tables 1-2.

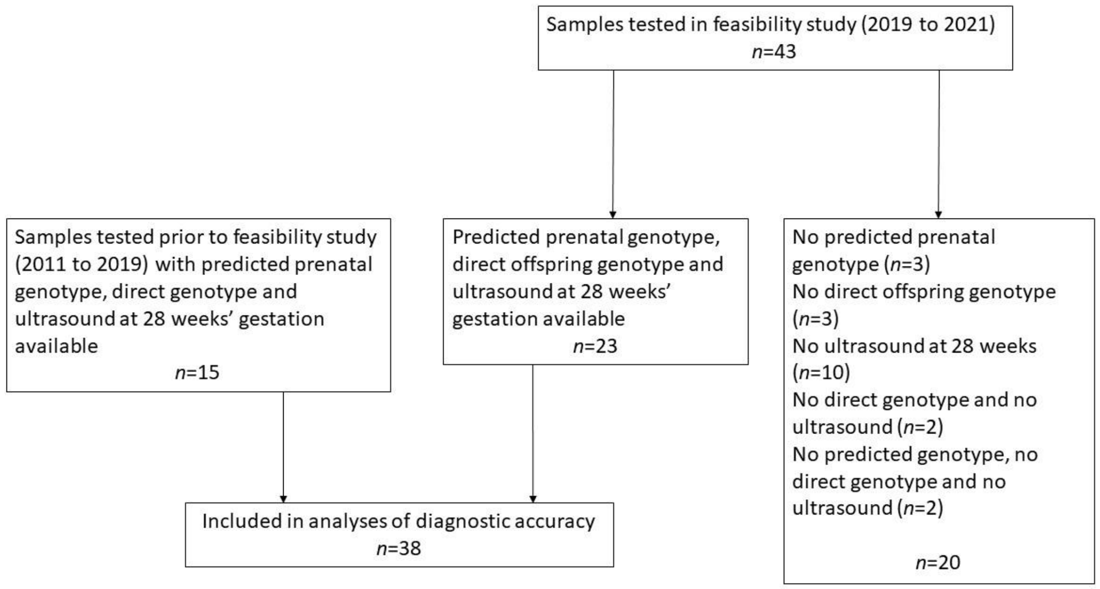

Supplementary Material References

1. Caswell RC, Snowsill T, Houghton JAL, et al (2020) Noninvasive Fetal Genotyping by Droplet Digital PCR to Identify Maternally Inherited Monogenic Diabetes Variants. Clin Chem 66(7):958–965. https://doi.org/10.1093/clinchem/hvaa104

2. Cole TJ, Freeman JV, Preece MA (1998) British 1990 growth reference centiles for weight, height, body mass index and head circumference fitted by maximum penalized likelihood. Statistics in Medicine 17(4):407–429. https://doi.org/10.1002/(SICI)1097-0258(19980228)17:4<407::AID-SIM742>3.0.CO;2-L
